# Automatic variable extraction from 3D coxal bone models for sex estimation using the DSP2 method

**DOI:** 10.1007/s00414-024-03301-4

**Published:** 2024-08-05

**Authors:** Michal Kuchař, Anežka Pilmann Kotěrová, Alexander Morávek, Frédéric Santos, Katarína Harnádková, Petr Henyš, Eugénia Cunha, Jaroslav Brůžek

**Affiliations:** 1 Department of Anatomy, Faculty of Medicine in Hradec Králové, Charles University, Šimkova, 870, Hradec Králové, 500 03 Czech Republic; 2 Department of Anthropology and Human Genetics, Faculty of Science, Charles University, Viničná 7, Prague 2, 128 44 Czech Republic; 3 CNRS, Univ. Bordeaux, MCC – UMR 5199 PACEA. Bâtiment B8, Allée Geoffroy Saint Hilaire, Pessac Cedex, CS 50023, 33615 France; 4 Institute of New Technologies and Applied Informatics, Faculty of Mechatronics, Informatics and Interdisciplinary Studies, Technical University of Liberec, Studentská 1402/2, Liberec, 461 17 Czech Republic; 5 Department of Life Sciences, Centre for Functional Ecology (CFE), Laboratory of Forensic Anthropology, University of Coimbra, Calçada Martim de Freitas, Coimbra, 3000-456 Portugal; 6 Instituto Nacional de Medicina Legal e Ciências Forenses, IP., Lisboa, Portugal

**Keywords:** Automatic variables extraction, DSP2 method, Sex estimation, Surface models, Forensic anthropology

## Abstract

Thanks to technical progress and the availability of virtual data, sex estimation methods as part of a biological profile are undergoing an inevitable evolution. Further reductions in subjectivity, but potentially also in measurement errors, can be brought by approaches that automate the extraction of variables. Such automatization also significantly accelerates and facilitates the specialist’s work. The aim of this study is (1) to apply a previously proposed algorithm (Kuchař et al. 2021) to automatically extract 10 variables used for the DSP2 sex estimation method, and (2) to test the robustness of the new automatic approach in a current heterogeneous population. For the first aim, we used a sample of 240 3D scans of pelvic bones from the same individuals, which were measured manually for the DSP database. For the second aim a sample of 108 pelvic bones from the New Mexico Decedent Image Database was used. The results showed high agreement between automatic and manual measurements with rTEM below 5% for all dimensions except two. The accuracy of final sex estimates based on all 10 variables was excellent (error rate 0.3%). However, we observed a higher number of undetermined individuals in the Portuguese sample (25% of males) and the New Mexican sample (36.5% of females). In conclusion, the procedure for automatic dimension extraction was successfully applied both to a different type of data and to a heterogeneous population.

**Supplementary Information:**

The online version contains supplementary material available at 10.1007/s00414-024-03301-4.

## Introduction

In forensic anthropology, biological sex estimation is one of the four elements that make up an individual’s biological profile, and has an important place in identifying an individual, sometimes halving a list of suspects. Skeletal sex estimation relies on morphological and metric dimorphic traits and uses classification tools [1, 2].

The main factors that influence the estimation of sex based on morphological characteristics are the observer’s subjectivity and experience, and frequent inconsistency in the evaluation of morphological characters. Nevertheless, morphoscopic analysis can yield valuable information. Metric methods, on the other hand, reduce the subjectivity typical of visual sex assessment. Most importantly, morphometric methods provide statistical parameters that are used to make a final decision [3, 4], enabling quantification of the results, which is beneficial in forensics.

It is generally accepted that the pelvis is the most suitable part of the skeleton for biological sex estimation (e.g. [5, 6]), followed by the long bones and the skull. One well-known and widely discussed tool for biological sex estimation is Probabilistic Sex Diagnosis 2 (DSP: Diagnose Sexuelle Probabiliste [7]), which has been tested and applied worldwide. DSP2 is a population non-specific method that uses 10 pelvic measurements based on a large and heterogeneous sample of the human metapopulation from four continents, and is grounded on linear discriminant analysis (LDA). Dedicated software is freely available (http://projets.pacea.u-bordeaux.fr/logiciel/DSP2/dsp2.html) to automatically estimate the sex of *os coxae* with varying degrees of bone preservation with a 95% probability of correct classification [8, 9]. The DSP2 method has been validated in various dry bone samples of known age and sex [10–16], and in the virtual environment on 3D models from CT (computed tomography) scans of living individuals [10, 17, 18]. Likewise, the interchangeability of DSP2 variables measured directly on dry bones, and on 3D models from CT and surface scanners, has been demonstrated [19–21].

Although DSP achieves excellent results, with a reliability exceeding 95% accuracy for sex estimation [22], a few exceptions have occurred. A higher number of undetermined individuals in a Mexican sample and in others has systematically occurred [11, 16]. Another example is the high misclassification rate in the Brazilian sample (9.4% of error for males and 14% for females) [14]. However, high misclassification was not confirmed by another study of the same population, with an error rate of 0.4% (de Almeida et al. 2020).

The success of sex estimation depends on the magnitude of sexual dimorphism in any given population [7], and not only on the method itself. Besides, varying accuracy may be due to inaccurate or incorrectly performed measurements by the observer. DSP2 is robust enough to a reasonable measurement error [7, 21], giving a high number of accurate estimates, due among other things to the presence of undetermined individuals. When using the posterior probability value (PP) for correct classification of 0.95, undetermined individuals exist, but are absent when using the traditional posterior probability of 0.5. Individuals with a PP of around 0.5 have almost the same probability of being female or male, which is unsatisfactory. With a decrease in the dividing value for methods using parts of the skeleton other than the pelvis, the number of undetermined individuals increases significantly [8]. Some researchers have considered decreasing the posterior probability value for correct classification in order to achieve a better balance between the number of classified individuals and accuracy (e.g. [23, 24]).

In light of technical progress, computational approaches are gaining prominence. These approaches aim to assist experts in reducing the influence of subjectivity and measurement errors introduced by evaluators (especially in the case of less experienced experts). Additionally, they help speed up work and allow for the analysis of a larger number of individuals. Semi-automatic and automatic detection and the placement of anthropological landmarks have led to more robust sex estimation [25–27]. Our study builds upon a method that enables the automatic definition of anthropological landmarks on pelvic bones, based on a large dataset of CT scans (*n* = 200, Czech population). We evaluate the performance of this method/algorithm for sex estimation using DSP2 [26].

### In the present study, we aimed to

1. Validate the previously proposed algorithm on a set of surface scans of pelvic bones from three different osteological collections. The type of data and its acquisition are different from those in the original study.
2. Test the robustness of the automated method by applying variable extraction on bones outside the DSP2 reference sample (forensic CT images of the current population).

## Material

Given the study’s distinct goals, we employed two sub-samples. The first sub-sample (validation) was used to assess the agreement between automatically calculated dimensions (for the DSP2 method) and measurements taken directly on dry bones. The second sub-sample (application) was used to test the automatic dimension extraction on a population distinct from the original DSP2 reference group. Table 1 provides the origin and distribution of individuals across both sub-samples.

### Validation sample

The sample contains a total of 240 3D scans of pelvic bones of males and females (some individuals provided both pelvic bones, i.e. some pelvic bones came from the same individual) from three osteological collections. The first collection originated in Portugal: the Coimbra Identified Skeletal Collection (CISC) housed at the University of Coimbra (19th -20th century) [28]. The second is the Simon Identified Skeletal Collection, housed at the Laboratory of Prehistoric Archaeology and Anthropology of the University of Geneva, Switzerland (19th -20th century) [29]. The third is from Lithuania: the Lithuanian osteological reference collection located at the State Forensic Medical Service, Vilnius (20th century) [30]. All the selected pelvic bones were part of an extensive dataset of pelvic bones whose dimensions were used in the development of the DSP2 method [7, 22].

### Application sample

The automatic variable extraction procedure was applied to a sample of 108 pelvic bones (56 males, 52 females) from the New Mexico Decedent Image Database (NMDID) [31]. We selected this database as it provides forensic data from a heterogeneous population, with clearly defined CT settings [31, 32]. All the individuals are 20th century births (1921–1994). Permission to analyze the CT scans of this present-day sample was provided by each person’s next of kin to the NMDID developers.

**Table 1:** Composition of validation (osteological collections) and application (CT image database) samples

	Validation sample		
	Males	Females	Total
Portugal (POR)	56	46	102
Lithuania (LIT)	30	38	68
Switzerland (SWI)	40	30	70
Total	126	114	240
	Application sample		
New Mexico Decedent Image Database (NM)	56	52	108

## Methods

### Data acquisition

All skeletal remains used in the validation sample were digitized with an HP 3D Structured Light Scanner PRO S2 or S3 surface scanner, and post-processed in integrated David LaserScanner v.3.10.4 software. The whole surface of the *os coxae* was scanned. To ensure that the models’ surfaces are closed and there are no mesh errors present we used automated Screened Poisson [33] surface reconstruction followed by Quadratic Edge Collapse Decimation. The reconstructed surfaces were then converted into .mha volumetric data. This volumetric data then underwent further analysis as stated in Bone shape registration.

CT whole-body-scans data for the application sample from the NMDID meets the following criteria: known sex, no decomposition, age 20+, torso protocol, and soft tissue reconstruction with 1 mm slice thickness/0.5 mm overlap. Scans with bone trauma were excluded. A combination of semi-automatic (GraphCut, TotalSegmentator) and manual (MITK-Workbench) segmentation was used to extract pelvic bone geometry from the CT scans. We then converted these geometries into .stl format, followed by conversion to .mha volumetric data, ensuring a consistent methodological approach with the surface scanner data.

### Bone shape registration

Each pelvic bone is unique, of a different size and shape, making automatic measurement difficult. However, all the bones are anatomically and topologically equivalent, allowing us to find the point correspondence between two shapes. In other words, it is possible to reversibly morph the examined bone into another, under a suitable class of transformation maps and similarity metrics. These transformation maps were computed using the Symmetric normalization registration method (SyN) [34] with previous rigid and affine transformations to roughly align the samples (‘demons’ metrics, ANTs registration library [35]) with parameters set up as stated in the authors’ previous work [26, 36]. We used the right and the left pelvic bone template from a previous study [26] as the morphing template. Finally, pelvic bones from both sub-samples were geometrically aligned to this template model and visually inspected to find any errors. A correlation between two shapes was applied in order to identify errors after the registration step. The correlation drops promptly to 0, when accounting for a mesh error or non-matched shapes. After bone registration, the pelvic bones are shape-normalized and ready for the next step, automatic measurements taken to apply the DSP2.

### Placement of landmarks on the template and variables measurements

**Fig. 1:**
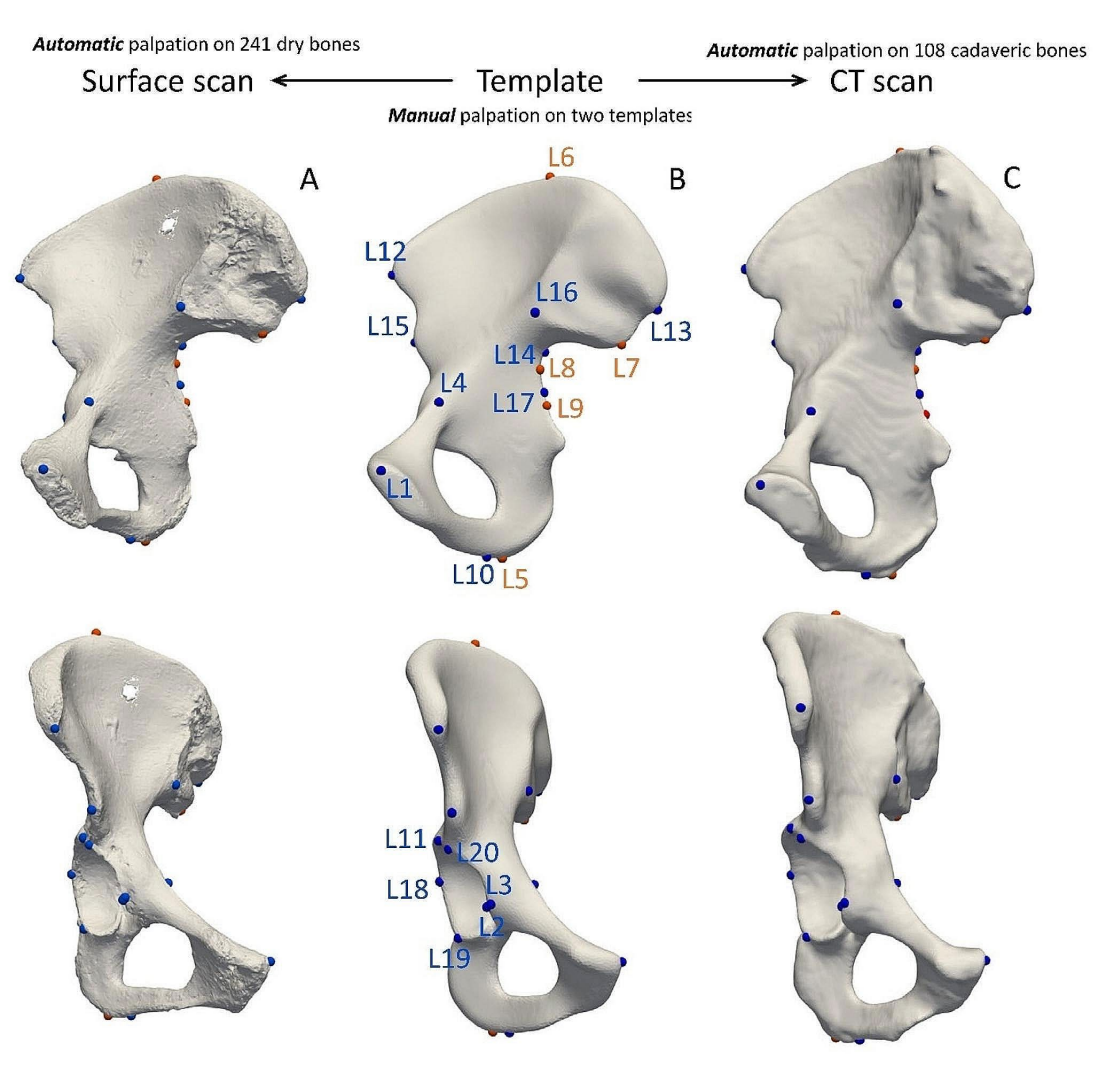
Seeding landmarks from a template bone to the dataset (view from different sides). **A** – an example of a surface scan of a dry pelvic bone (Coimbra sample, female), all landmarks placed automatically; **B** – Right template, landmarks in blue are manually placed/palpated, landmarks in orange are artificially placed; **C** – an example of a CT scan (New Mexico sample, male), all landmarks placed automatically

We mapped the landmarks corresponding to pelvic bone measurements proposed by the DSP2 to the right and left template bone under the guidance of the original method author (JB) [7, 22]. The reference landmarks (L1–L4, L7–L20) palpated to the templates defined nine DSP distances (D1,2,4–D10), see Table 2. Landmarks L5 and L6 (for D3 distance) were automatically calculated for each bone, as the landmarks that define the maximal length cannot be anchored to a specific spot on the template. Here, the superficial mesh of each model was evenly covered by 30,000 points and the maximal distance between two points was calculated (using *brute-force)*. Transfer of distance 4 (greater sciatic notch height - IIMT) from a real to a virtual environment proved to be challenging. We revised the original distance definition and suggested an alternative by projecting three points (L7–L9), to the greater sciatic notch and posterior inferior iliac spine. This process enabled us to use Heron’s formula to measure the height of the greater sciatic notch. Measured distances D3 and D4 are shown in detail in Fig. 1. Knowing the mutual relationship between the template and original bones, we then seeded all the landmarks into our dataset (both sub-samples). Finally, every bone was covered by a set of landmarks, ready for calculation of Euclidean distances.

**Table 2:** Reference landmarks and distances definitions

L1	The most superior and medial point on the pubic symphysis	D1 = ||L1 – L2||	Acetabulo-symphyseal pubic length (PUM)
L2	Anterior border of the acetabular rim at the level of the lunate surface		
L3	The most lateral point on the acetabular rim	D2 = ||L3 – L4||	Cotylo-pubic breadth (SPU)
L4	A point on the medial margin of the pubic bone; at the level of L3		
L5	The most inferior point of the os coxae	D3 = ||L5 – L6||	Maximum pelvic height (DCOX)
L6	The most superior point of the os coxae		
L7	Posterior superior iliac spine	D4 (see Fig. 2)	Depth of the greater sciatic notch (IIMT)
L8	Dorsal part of greater sciatic notch		
L9	Ventral part of greater sciatic notch		
L10	The most anterior and inferior point on the ischial tuberosity	D5 = ||L10 – L11||	Post-acetabular ischium length (ISMM)
L11	The furthest point on the acetabular margin from L10		
L12	Anterior superior iliac spine	D6 = ||L12 – L13||	Iliac breadth (SCOX)
L13	Posterior superior iliac spine		
L14	The deepest point of the greater sciatic notch	D7 = ||L14 – L15||	Spino-sciatic length (SS)
L15	Anterior inferior iliac spine		
L16	The contact point of the arcuate line and the auricular surface	D8 = ||L15 – L16||	Spino-auricular length (SA)
L17	The midpoint of the anterior portion of the greater sciatic notch	D9 = ||L17 – L18||	Cotylo-sciatic length (SIS)
L18	A point on the lateral border of acetabulum; at the level of L17		
L19	The most inferior point on the acetabular rim in the longitudinal axis of ischium	D10 = ||L19 – L20||	Vertical acetabular diameter (VEAC)
L20	The most superior point on the acetabular rim; in the longitudinal axis of the ischium		

**Fig. 2:**
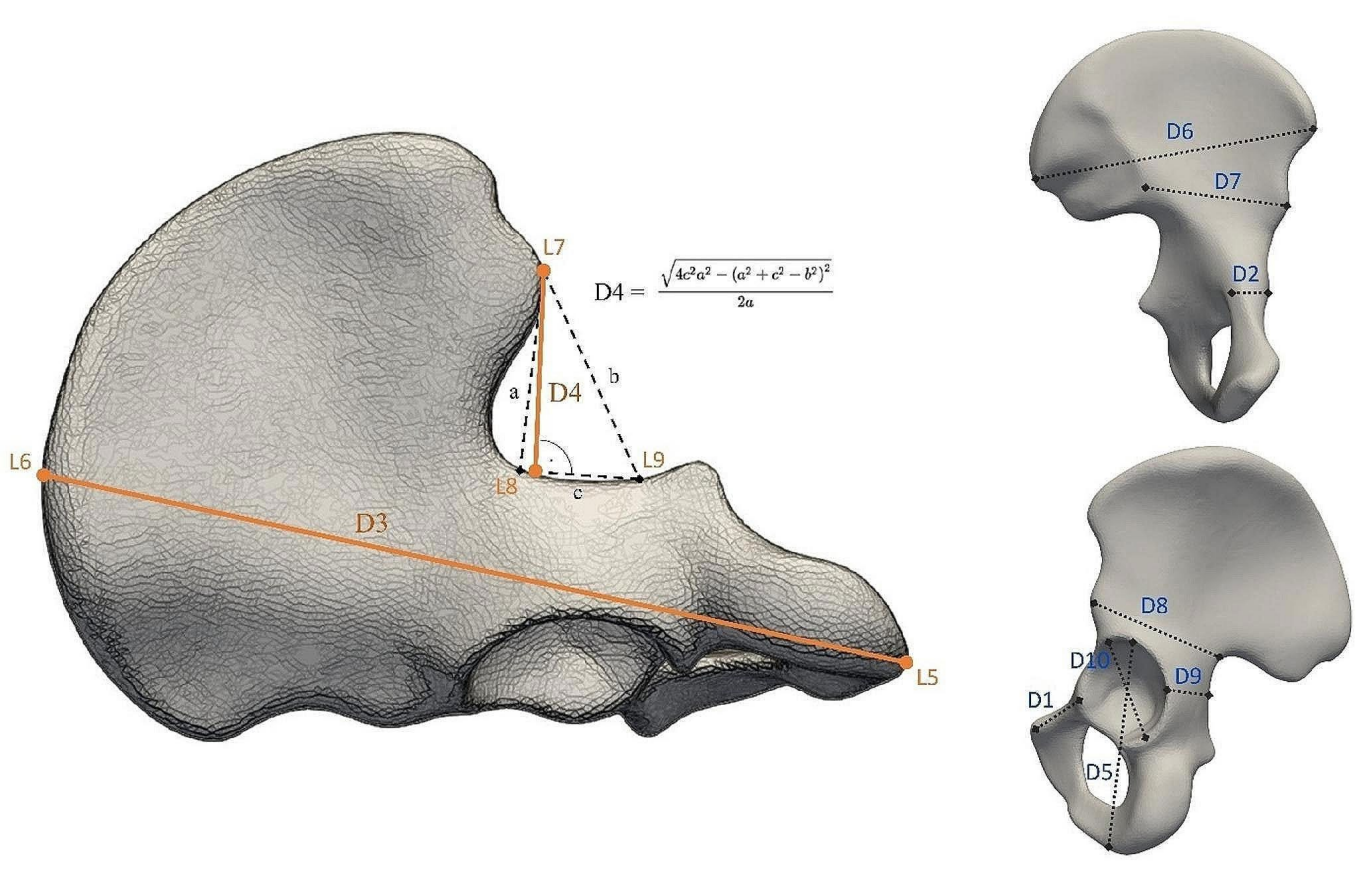
Measured distances on pelvic bones. Automatic measurement of D3 (L5–L6) is based on the model’s two most distant points, and measurement of D4 (L7–L9) on Heron’s formula

Automatically calculated measurements in the validation sample (3D models of pelvic bones identical to those used to develop the DSP) were then compared to those measured by one of the authors (JB) of the DSP method directly on corresponding/matching dry bones. To verify the accuracy of the measured dimensions within each type of data (dry and virtual), i.e. the agreement between them, we calculated mean error (the arithmetic mean of the difference between measurements), Lin’s concordance correlation coefficient (Lin’s CCC) [37], the technical error of measurement (TEM), and the relative TEM (rTEM) expressed as a percentage [38]. All analyses were performed using R-project 4.3.3 [39].

### Sex classification using the measured distances

The distances D served as an input into the DSP2 classification algorithm. As IIMT is complicated in definition and challenging to measure virtually [19, 20], we estimated the sex of individuals using sets of nine and ten distances respectively (i.e. with and without the IIMT). The probability of being a male or female was assessed according to the linear discriminant analysis and posterior probabilities. For reliable sex estimation, a posterior probability greater than 0.95 was considered to be the classification threshold; below this value, estimates are considered indeterminate [7].

The resulting sex diagnoses (F – female, M – male, I - indeterminate) in both validation and application samples were subsequently compared to the known sex of individuals.

### Population dissimilarities

To test whether there are any population differences in the sample, the following statistical approaches were applied. To test the presence of differences in variability among our studied populations, Bartlett’s test was applied to the population-samples’ variances of each measurement. To test for statistically significant differences in mean values among population samples, ANOVA and post-hoc pairwise t-tests with Bonferroni correction of the p-value were used. The Bonferroni correction [40] was applied for 60 pairwise tests. The adjusted p-value for the significance threshold was taken to be 0.00083. To emphasize inter-population differences, linear discriminant analysis (LDA) was performed. We plotted all individuals on the first and second discriminant axes and drew populational ellipses around 90% of the variance of each population.

## Results

The raw data from the automatically extracted and dry bone measurements are available as Supplementary materials (Suppl. 1–4). The Python and R scripts, along with comments, have been provided to other researchers as well (DOI 10.6084/m9.figshare.26232398).

### Population dissimilarities

The dataset consisted of 166 females and 182 males; measurements were analysed for both the left (205) and right (143) sides. The numerical values representing the mean measurement for each dimension within the respective population are presented in Table 3.

**Table 3:** Mean values (in mm) by sex and population sample for each variable

	LIT f	LIT m	POR f	POR m	SWI f	SWI m	NM f	NM m
D1 (PUM)	75.41	72.62	69.96	68.52	71.95	71.11	74.12	74.35
D2 (SPU)	22.66	29.59	20.92	27.04	22.12	28.47	24.29	30.36
D3 (DCOX)	206.24	220.37	195.65	208.55	200.24	220.0	210.23	229.06
D4 (IIMT)	49.48	44.58	46.67	42.31	47.12	44.25	45.99	44.72
D5 (ISMM)	104.79	115.01	99.1	110.2	101.87	116.13	104.18	117.49
D6 (SCOX)	157.87	160.1	148.21	148.81	149.46	155.9	148.96	160.37
D7 (SS)	69.27	74.92	66.25	71.39	69.5	76.57	72.56	80.54
D8 (SA)	76.19	77.17	71.6	72.96	74.8	77.35	76.4	80.69
D9 (SIS)	34.84	38.19	33.05	36.5	34.9	38.92	35.51	40.82
D10 (VEAC)	52.66	57.58	50.47	55.81	51.76	58.7	52.2	59.12

**Fig. 3:**
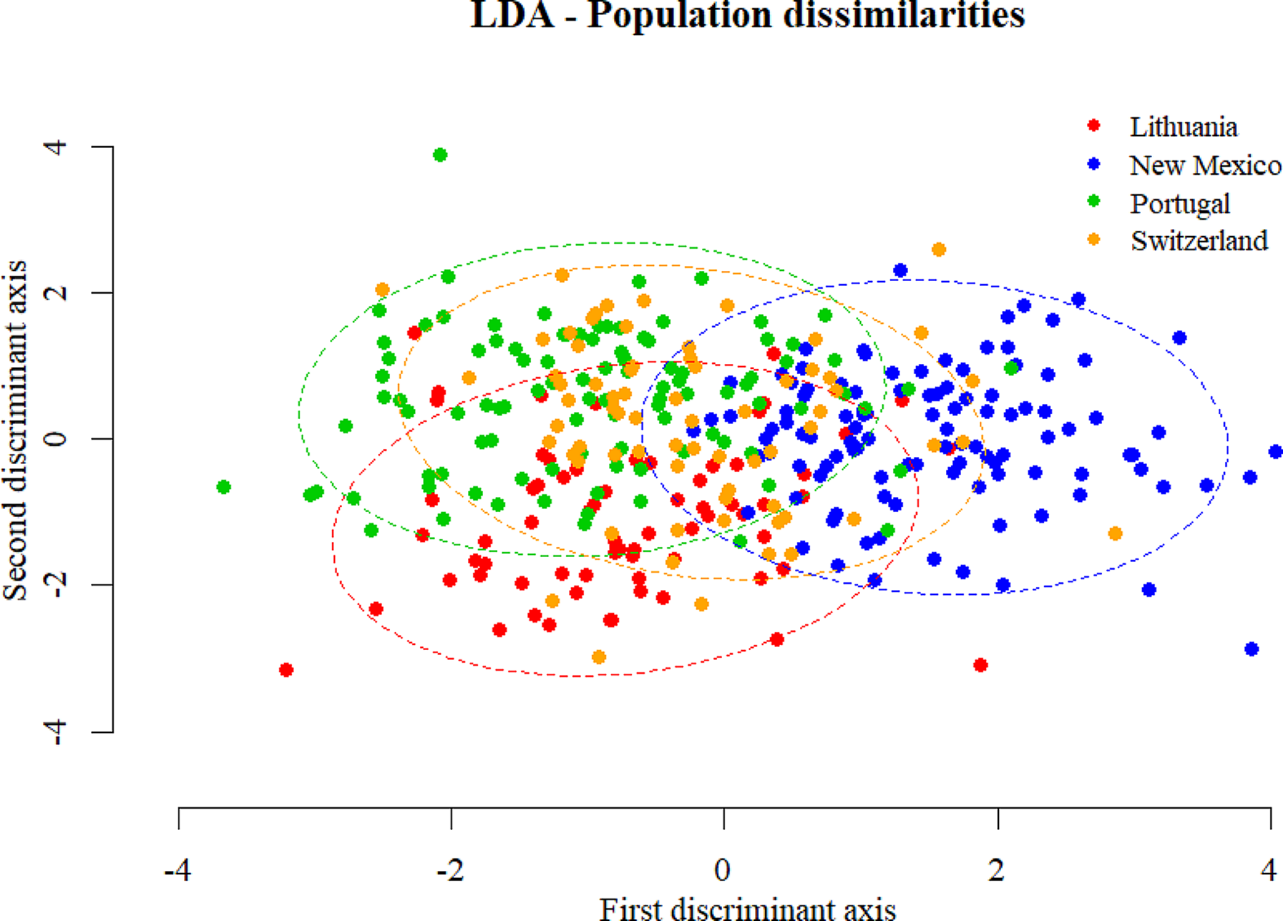
Dot plot depicting population dissimilarities pooled for both sexes. Dots represent individuals coloured by population, while ellipses depict 90% of the variability in each population. Multidimensional data are shown on the first and second discriminant axes from LDA

Bartlett’s test showed no statistically significant differences between population variances (p-val 0.1246) which implies that the intrapopulation variability is similar in all the studied populations. ANOVA results indicate that there are differences in mean values between populations (p-val < 2·10^− 16^). Pairwise t-tests with Bonferroni correction showed statistically significant differences, mainly for the New Mexican population (p-val < 0.00083) differing from other populations in D1–D3 and D5–D10. The extent of population differences is shown in Fig. 3, which utilizes the first and second discriminant axes from LDA.

### Concordance between dry bone and automatically extracted measurements

**Fig. 4:**
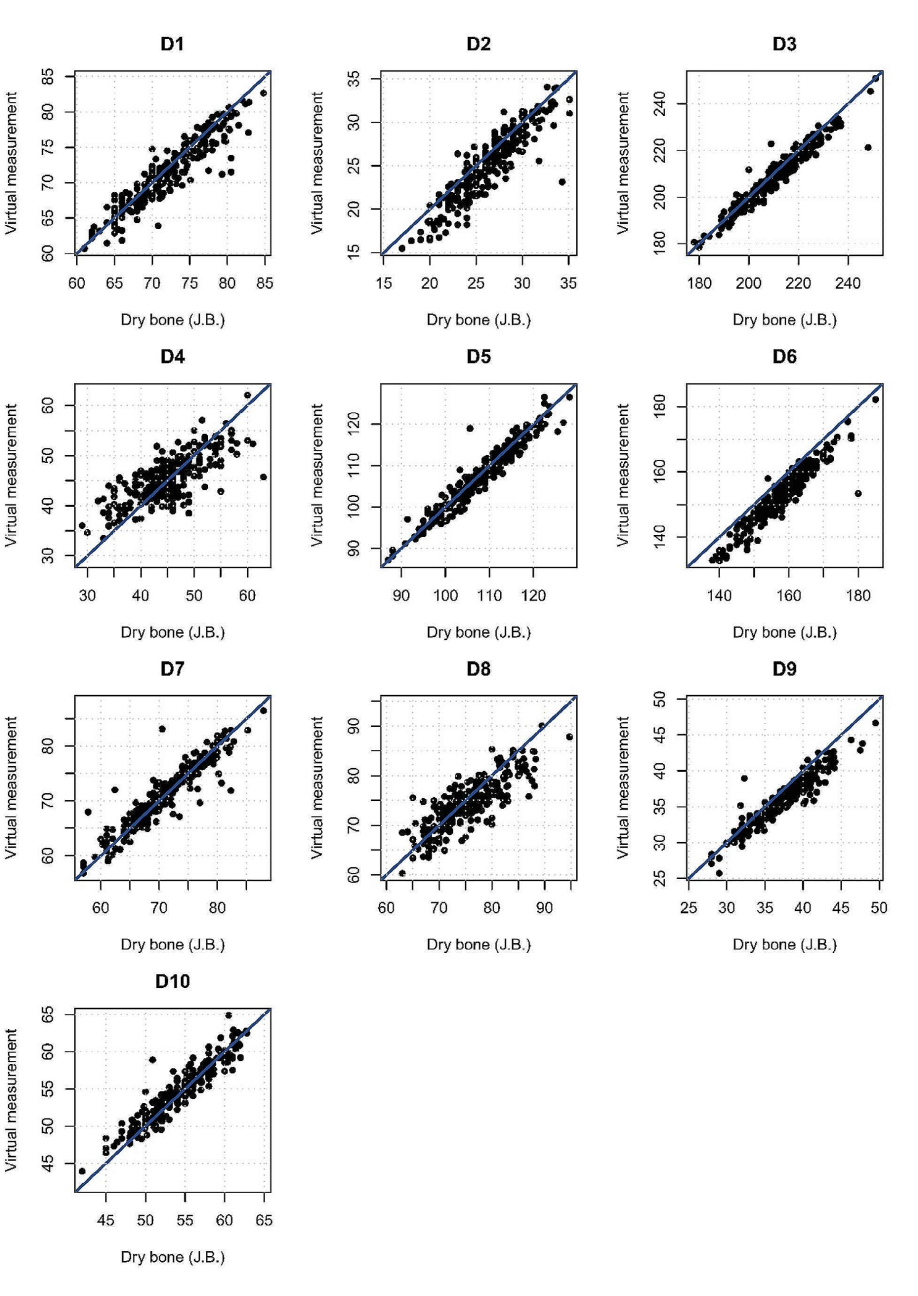
Biplots values between measurements taken on dry bone by one observer (JB) and measurements automatically extracted by the virtual model. The blue line is the line of perfect agreement: any point lying exactly on that line has the same measurement for both variables

The concordance between the measurements taken on dry bones (the validation sample) and the measurements extracted automatically is shown in Fig. 4. We found a few individuals exhibiting a high error for some variables.

The results of mean error, Lin’s concordance coefficient, TEM, and rTEM comparing the agreement between the dimensions measured on dry bones and the automatically extracted dimensions in the virtual environment (the validation sample) are shown in Table 4. Negative values for mean errors indicate smaller automatically extracted variable values compared to values measured on dry bones. Most of the distances are under 5% of rTEM, except SPU-D2 (5.9%) and IIMT-D4 (6.6%). The results show excellent correlation (Lin’s CCC > 0.8) according to Altman [41] for all distances (except for D4). Distances D3, D5, D7, and D10 ranged between 0.92 and 0.96. Several variables (D2, D6, D9) show consistently lower values with the virtual model than with the measurements taken on the dry bone.

**Table 4:** Summary of the agreement between automatically extracted and dry bone measurements for each variable

	Mean error	Lin’s CCC	TEM	rTEM
D1 (PUM)	-0.7	0.91	1.41	1.97
D2 (SPU)	-1.29	0.85	1.52	5.9
D3 (DCOX)	-2	0.95	2.79	1.34
D4 (IIMT)	0.59	0.7	2.98	6.6
D5 (ISMM)	-0.32	0.96	1.54	1.43
D6 (SCOX)	-5.06	0.82	4.11	2.64
D7 (SS)	0.19	0.92	1.58	2.22
D8 (SA)	-1.1	0.8	2.52	3.35
D9 (SIS)	-1.32	0.88	1.3	3.54
D10 (VEAC)	0.34	0.94	1.02	1.87

Mean error (in mm); Lin’s CCC – concordance correlation coefficient; TEM – technical error of measurement (in mm); rTEM – relative technical error of measurement (as a percentage)

### Sex estimation

By integrating measurements D1 through D10, the DSP2 software facilitated an analysis of sexual dimorphism across the given populations (from both the validation and application samples).

Results of sex estimation with all ten variables for all population samples are presented in Table 5. The overall accuracy was excellent (only one misclassified individual), with a classification error rate of a mere 0.3% across all examined samples. Given the 0.95 posterior probability provided by DSP2, the overall rate of indeterminate cases was reported at 11.5%, with some variation across populations. The Lithuanian and Swiss samples demonstrated the lowest indeterminacy rates, with a high success rate in sex estimation. The Portuguese and New Mexican samples showed slight variations in indeterminate rates (15 POR, 19 NM) while maintaining high accuracy.

**Table 5:** Sex estimation by DSP2 software for all 10 variables across both samples (validation and application)

		Estimated sex		
Population	True sex	F	I	M
**LIT**	F	37	1	0
	M	0	3	27
**POR**	F	45	1	0
	M	0	14	42
**SWI**	F	29	0	1
	M	0	2	38
**NM**	F	33	19	0
	M	0	0	56

LIT – Lithuanian; POR – Portuguese; SWI – Swiss; NM – New Mexican

Table 6 shows the results of sex estimation across all samples without the IIMT variable. Removing the IIMT led to an increase in the indeterminate rate to 13%. The classification error rate remained low at 0.3%, with the same distribution as with IIMT included.

**Table 6:** Sex estimation by DSP2 software without the IIMT variable (for each population sample)

		Estimated sex		
Population	True sex	F	I	M
**LIT**	F	37	1	0
	M	0	3	27
**POR**	F	45	1	0
	M	0	14	42
**SWI**	F	28	1	1
	M	0	2	38
**NM**	F	28	24	0
	M	0	0	56

LIT – Lithuanian; POR – Portuguese; SWI – Swiss; NM – New Mexican

## Discussion

The human pelvis is the most suitable bone for adult sex estimation because of its marked sexual dimorphism, which results mainly from selective constraints on males and females imposed by locomotion and childbearing. It is very likely that the current pattern of pelvic sexual dimorphism appeared in early modern humans approximately 100–150 ky ago [42, 43]. Despite these distinctions, there is a universally common pattern to the shape of the pelvic bone that enables the automation of seeding anthropological landmarks and capturing of its metrics and morphological characteristics. This approach is not yet common, but the first studies on this topic are beginning to appear in the anthropological literature. Based on previous work on facial morphology [44], Mbonani [27] applied the procedure of automatic landmarking to the pelvic complex; this study didn’t proceed to distance measurements, but stated an increase in objectivity and reliability. Using a similar technique, we moved from the pelvic girdle to separate pelvic bones, as they are more common in archaeologic and forensic contexts.

### Operator induced variability (and its removal)

Direct measurement of the 10 variables used in DSP2 by the operator, on bones or on 3D models from surface scans and CT, shows variability and is burdened by intra- and inter-observer errors (e.g. [12, 45, 46]). The use of automatic variable measurement completely eliminates this measurement variability.

When comparing automatic measurements with manual measurements performed on dry bones, we noted a high degree of agreement. In our experience, the registration algorithm is capable of recognizing prominent anatomical structures such as iliac spines, the deepest point of the sciatic notch or the margins of the acetabular fossa. Moreover, with precise definition, the absolute length of the bone can be measured error-free by calculating the Euclidean distance between the most distant points on a model. We consider this a significant step forward, as it is the first time the automatic process has bypassed template marking.

The greatest discrepancy from the original measurement was found for the automated measurement of IIMT. Here, we faced the same problem presented in the work of Braun [20], who also detected high rTEM for the IIMT measurement. Similarly to their paper, we adapted this variable for our purposes. In our case, we kept one point in the location of the posterior inferior iliac spine; we then introduced two new landmarks that form the apexes of a triangle, with the IIMT as the triangle’s height. The redefinition of this measurement was shown to be practical, as it helped in further sex estimation. The SCOX measurements showed consistently lower on virtual bones than on measurements taken on dry bones; this was surprising, given its clear definition and good rTEM values presented in a related study [19]. We attribute this to the loss of details (possibly osteophytes) around the posterior superior iliac spine as a result of smoothing the surface scans.

### Sex estimation accuracy with automatic variable extraction

Applying the distances to the DSP2 software demonstrated excellent concordance with the actual sex of individuals. Our overall success rate (88.2% correctly assigned, 11.5% indeterminate, 0.3% errors) aligns with studies validating the DSP2 method on dry bone samples [10–16]. Individuals classified as indeterminate mostly have PP in the range of 0.500 to 0.949. Thus, from the perspective of classical classification, they are diagnosed correctly, but with a value below the dividing threshold, and therefore do not contribute to the accuracy rate. We do not expect our method to surpass the performance of a highly experienced observer, who can adapt to any possible bone variations or deformations. However, compared to traditional measurements, the registration algorithm shines in analyzing large amounts of data. While meeting the difficulties with alternate landmark settings, we were able to perform multiple test measurements on all samples within a few minutes.

The New Mexico sample included a current population and different imaging technique (CT) to our dataset. With the same template and set of landmarks, we automated the measurement of segmented pelvic bones and used these measurements with the DSP2 software. The results remained promising, correctly identifying the sex of all determined individuals. However, we observed an increase in the number of indeterminate female cases (36.5%), while all males were correctly assigned. The retrospective visual control did not confirm any errors in the landmark setting. A higher number of indeterminate cases was also found in studies by Sánchez-Mejorada (19%) and de Almeida (22.5%) [11, 16] for males. Explanation is difficult and may relate to inter-populational variability or an insufficient sample. For the automatic measurements, we used pelvic bones from four collections (Portuguese, Swiss, Lithuanian and New Mexican). There were no differences between populational variances, but the New Mexican individuals presented significantly higher mean values at most distances. This altered the DSP2 performance, leaving some of the females under the 95% level of posterior probability. The effects of population variability, imaging techniques, and possible secular changes in the innominate are targets for the next work.

### Disadvantages and study limits

As previously discussed, measuring IIMT presents challenges regarding its definition and the placement of points. To evaluate the cost-effectiveness of modifying the measurement method, we conducted two sets of sex estimation tests across all populations. These tests were performed once including IIMT (resulting in ten distances) and once excluding IIMT (resulting in nine distances). In our opinion, the information contained within this diameter is strong enough to be involved. Despite having the lowest Lin’s concordance coefficient among the measurements, incorporating this value slightly increased the correctness of sex estimation and reduced the number of cases where sex could not be determined.

We are aware of several limitations to our method. Choosing of right template is crucial for the measurement process. In this study, we selected a smoothed version, which helped cover individual bones’ minor irregularities; some details can however be lost as the registration algorithm may not accurately identify detailed structures like osteophytes. Moreover, the quality of the surface or CT scans must be sufficient to align properly with the template. While we can automatically correct minor defects in the surface mesh, we still expect the end user to have some skill in photogrammetry or segmentation. Despite the demonstrated interchangeability of DSP2 variable measurements across different imaging techniques, minor discrepancies can arise due to varying image modality settings; these discrepancies are unlikely to affect the performance of the registration algorithm but may impact the accuracy of sex determination provided by DSP2.

### Future work

Encouraged by the promising results, we aim to develop a fully automatic application for sex estimation, making it more accessible and user-friendly for forensic practitioners and researchers. Additionally, we will explore the possibilities of automatic variable extraction on bone fragments, addressing the challenges of incomplete skeletal remains.

## Conclusion

This study has successfully demonstrated the efficacy of the DSP2 method in sex estimation of coxal bone using automatic variable extraction. Moving from manual landmarking to an automated process, which can be considered a major step forward, we provide a validated approach that combines the strengths of DSP2 with the precision and objectivity of computational techniques. This method can analyze a single bone or expand to a dataset that includes a broader range of individuals, while the impact of human subjectivity and the intra- and inter-observer errors is decreased. This shows promise not only in forensic anthropology but also in archaeological contexts where rapid and accurate sex estimation is critical.

## Electronic supplementary material

Below is the link to the electronic supplementary material.

Supplementary Material 1

Supplementary Material 2

Supplementary Material 3

Supplementary Material 4
