# Correction to: Automatic variable extraction from 3D coxal bone models for sex estimation using the DSP2 method

**DOI:** 10.1007/s00414-024-03311-2

**Published:** 2024-08-12

**Authors:** Michal Kuchař, Anežka Pilmann Kotěrová, Alexander Morávek, Frédéric Santos, Katarína Harnádková, Petr Henyš, Eugénia Cunha, Jaroslav Brůžek

**Affiliations:** 1 Department of Anatomy, Faculty of Medicine in Hradec Králové, Charles University, 870, 500 03 Šimkova, Hradec Králové Czech Republic; 2 Department of Anthropology and Human Genetics, Faculty of Science, Charles University, Viničná 7, 128 44 Prague 2, Czech Republic; 3 CNRS, Univ. Bordeaux, MCC – UMR 5199 PACEA, Bâtiment B8, Allée Geoffroy Saint Hilaire, 50023, 33615 Pessac Cedex, CS France; 4 Institute of New Technologies and Applied Informatics, Faculty of Mechatronics, Informatics and Interdisciplinary Studies, Technical University of Liberec, Studentská 1402/2, 461 17 Liberec, Czech Republic; 5 Department of Life Sciences, Centre for Functional Ecology (CFE), Laboratory of Forensic Anthropology, University of Coimbra, Calçada Martim de Freitas, 3000-456 Coimbra, Portugal; 6 Instituto Nacional de Medicina Legal e Ciências Forenses, Lisboa, IP Portugal

**Correction to: International Journal of Legal Medicine (2024)**

10.1007/s00414-024-03301-4

The article was initially published with the author names inverted, where the family name was listed first instead of the given name. This has now been updated here.

The original article has been corrected.

